# Supplementary material for: Longitudinal field controls vector vortex beams in anisotropic epsilon-near-zero metamaterials
Source: Nat Commun. 2025 Apr 23;16:3807. doi: 10.1038/s41467-025-58088-8 (PMC12019337; doi:10.1038/s41467-025-58088-8)
Supplement: Supplementary file 1 — Supplementary Information [file 41467_2025_58088_MOESM1_ESM.pdf]

Supporting Information

Longitudinal field controls vector vortex beams in anisotropic epsilon-near-zero metamaterials

Vittorio Aita, Diane J. Roth, Anastasia Zaleska, Alexey V. Krasavin, Luke H. Nicholls, Mykyta Shevchenko, Francisco J. Rodríguez-Fortuño, and Anatoly V. Zayats

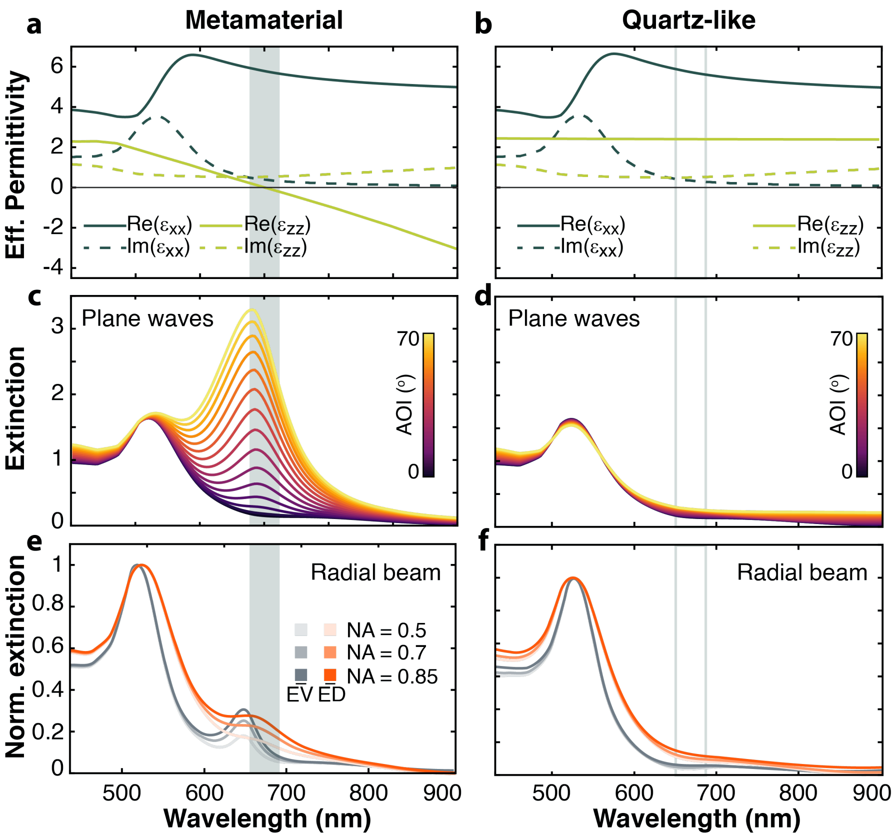

Supplementary Figure 1 Role of the epsilon near zero regime. (a,b) Effective permittivity components and (c–f) extinction spectra obtained with the sample illuminated by (c,d) *p*-polarised plane waves at angles of incidence (AOI) ranging from zero (normal incidence) to 70° and (e,f) a focused radial beam with increasing NA. The material permittivity represents (a,c,e) the metamaterial studied in this work and (b,d,f) a hypothetical anisotropic crystal with the exact same permittivity of the metamaterial, apart from the real part of its ε_z component, which is set to the extraordinary refractive index of quartz, taken from Ref. 1. The same as in Fig. 2, panels (e,f) show a comparison between evaporated (EV) and electrodeposited (ED) gold.

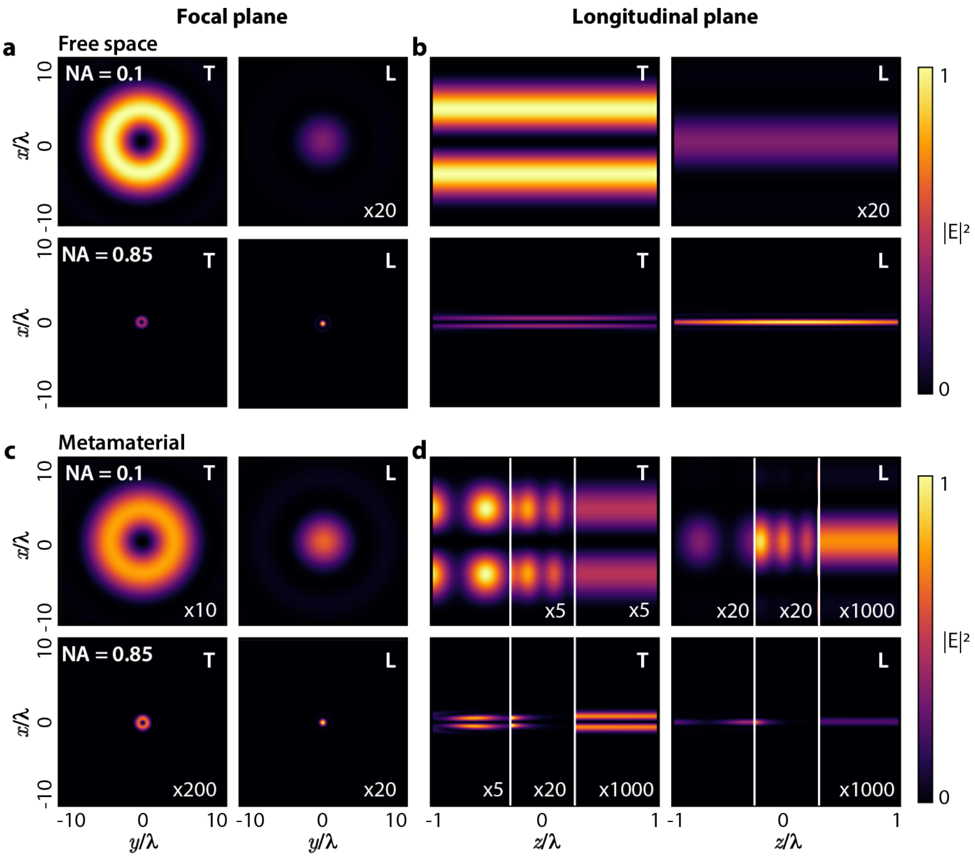

Supplementary Figure 2 Propagation and diffraction of a radial beam. Intensity distributions of the transverse (T) and longitudinal (L) electric field components at a wavelength of $\lambda_{\mathrm{ENZ}} = 660$ nm of a focused radial beam propagating through (a,b) free space and (c,d) the nanorod metamaterial. Cross sections are taken in (a,c) the focal plane and (b,d) across the *xz*-plane in the case of (upper row of each panel) weak (NA=0.1) and (lower row of each panel) strong (NA=0.85) focusing. The metamaterial parameters are as in Fig. 1c).

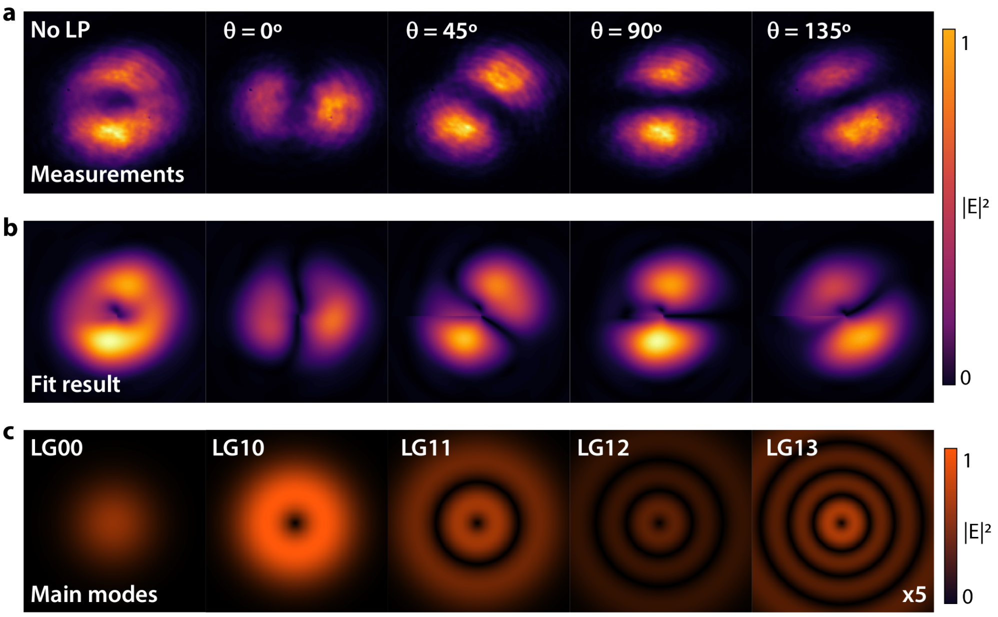

Supplementary Figure 3 Fitting of the modal content of a radial beam. (a) Experimental intensity distributions of a radial beam propagating through the metamaterial and focused with an NA = 0.7 objective without and with a linear polariser for several axis orientations. (b) Intensity distributions obtained from the fitting procedure (Eqs.6,7) corresponding to the conditions in (a). (c) Normalised intensities of the main LG modes contributing to the beam modal content, according to the fit results.

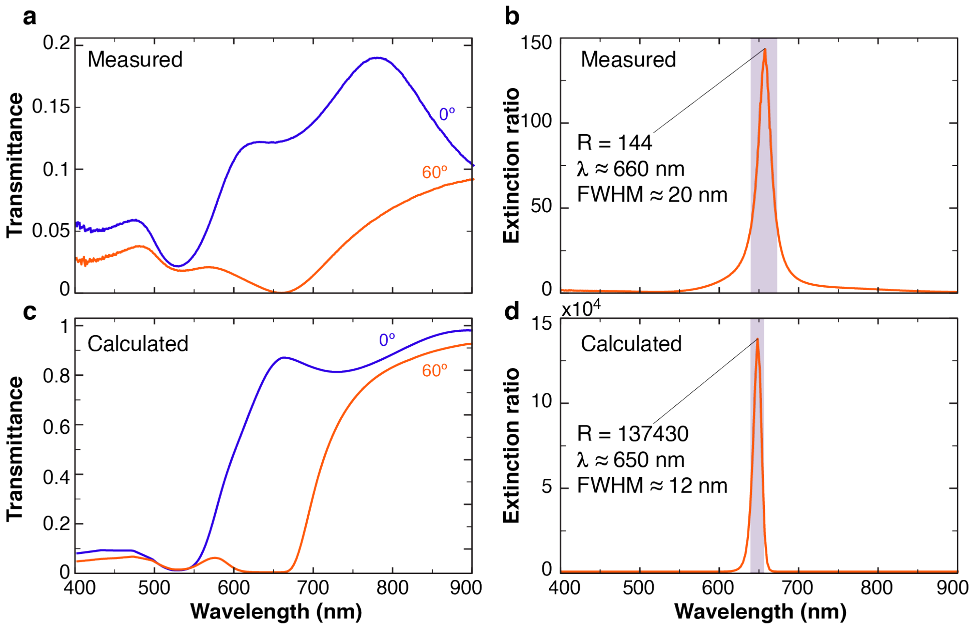

Supplementary Figure 4 polarization extinction spectroscopy. (a,b) Measured and (c,d) calculated (a,b) transmission spectra and (c,d) polarization extinction ratios (R) for *p*-polarised illumination at an angle of incidence of (blue) 0° and (orange) 60°. The shaded areas in panels (b,d) show the bandwidth as the FWHM of the peak.

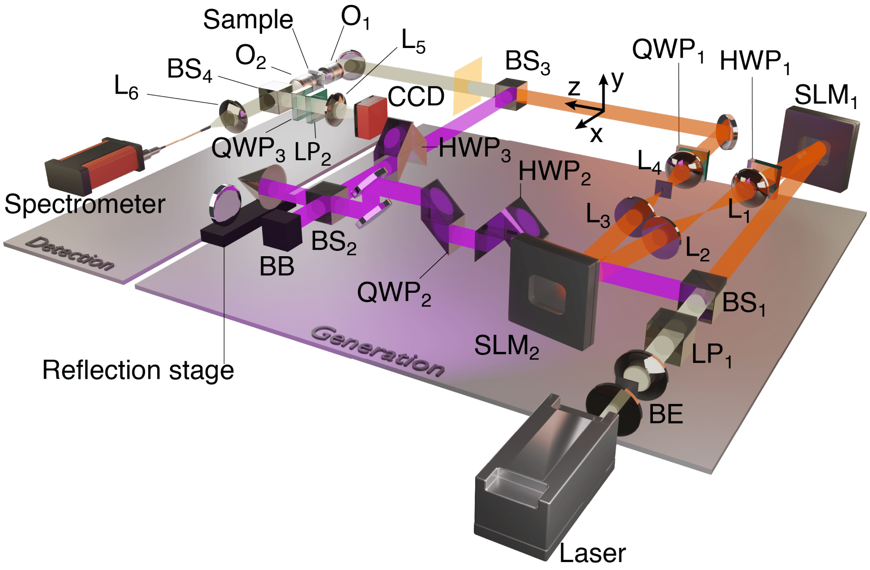

Supplementary Figure 5 Experimental setup. Schematic representation of the experimental setup for beam preparation (generation stage) and optical characterization (detection stage). The generation stage comprises two branches corresponding to (orange) monochromatic and (purple) broadband vector beams generation. Optical components are labelled as follows: BE - beam expander, LP - linear polariser, BS - beam splitter, SLM - spatial light modulator, L - lens, HWP - half waveplate, QWP - quarter waveplate, O - objective, BB - beam blocker. The focal lengths of the lenses are: $f_1 = f_2 = 150$ mm, $f_3 = f_4 = 200$ mm, $f_5 = f_6 = 200$ mm. The sample is mounted on a customised stage allowing full translation and rotation. The *x*-*y*-*z* reference frame follows the beam path and the plane indicated in orange represents the output of the generation stage

.
